# Determination of Bisphenol Compounds and the Bioaccumulation after Co-Exposure with Polyethylene Microplastics in Zebrafish

**DOI:** 10.3390/toxics12100702

**Published:** 2024-09-27

**Authors:** Moyong Xue, Ming Jia, Yuchang Qin, Jing Li, Ting Yao, Frédéric Francis, Xu Gu

**Affiliations:** 1 Feed Research Institute, Chinese Academy of Agricultural Science, Beijing 100081, China; moyong.xue@student.uliege.be (M.X.);; 2 Functional and Evolutionary Entomology, Gembloux Agro-Bio Tech, University of Liege, 5030 Gembloux, Belgium; frederic.francis@uliege.be; 3 Institute of Animal Science, Chinese Academy of Agriculture Sciences, Beijing 100193, China; 4 Beijing Institute of Food Control, Beijing 110108, China

**Keywords:** bisphenol compounds, microplastic, co-exposure, bioaccumulation

## Abstract

Knowledge regarding the combined toxicity mechanism of bisphenol compounds and microplastics (MPs) on organisms remains limited. In this study, we first developed an accurate and sensitive method to simultaneously quantify two bisphenol compounds and evaluate their accumulation and tissue distribution after co-exposure with MPs in zebrafish. Then, we determined the bioaccumulation potential of bisphenol A (BPA) and bisphenol S (BPS) in adult zebrafish in the absence and presence of MPs. Bisphenol compounds were found to accumulate in different tissues of zebrafish, with BPS showing lower accumulation levels compared to BPA. Importantly, we discovered that the presence of MPs could exacerbate the accumulation of bisphenol compounds in biological tissues. These findings highlight the enhanced bioavailability and risk posed by the co-exposure of bisphenol compounds and MPs, underscoring the need for further investigation into their combined environmental and biological health impacts.

## 1. Introduction

The endocrine-disrupting chemical bisphenol A (BPA) is nearly ubiquitous in natural environments [1]. In recent years, growing evidence has confirmed that it can bind to various hormone receptors and subsequently affect normal physiological processes, such as reproductive disorders, immune responses, and cardiovascular disease [2,3,4]. The adverse effects of BPA on aquatic organisms and ecosystem health have caused significant public concern [5,6]. For this reason, BPA has been banned in many products and has gradually begun to be replaced with alternatives, such as bisphenol S (BPS) [7]. However, their similar chemical structures and estrogenic activities may lead to similar physiological effects [8,9].

Microplastics (MPs), another global environmental issue, also severely threaten natural ecosystems and humanity [10]. Most importantly, MPs have emerged as a global concern, not only because of their ecotoxicological impacts but also because of their interactions with other pollutants [11]. Many studies have proved that MPs could interact with other pollutants owing to their strong hydrophobicity [12,13,14], causing further ecotoxicological impacts [15]. Furthermore, growing evidence suggested that MPs could interact with bisphenol compounds in aquatic environments and accumulate in exposed organisms, causing further negative effects [15,16,17]. The toxic effects of co-exposure to bisphenol compounds and MPs have been extensively studied, and it has been demonstrated that the presence of MPs could affect the bioavailability and toxicity of bisphenol compounds in organisms [12,16,17]. However, to the best of our knowledge, whether the presence of MPs could affect the accumulation and distribution of bisphenol compounds within organisms has been rarely investigated. Thus, there is an urgent need to investigate the bioaccumulation of bisphenol compounds after co-exposure with MPs in aquatic organisms.

At present, the determination methods for bisphenol compounds are mainly focused on biological human matrices [18,19,20], food products [21,22,23], and environmental media [24,25]. There are few methods to determine with a single analysis the concentrations of multiple bisphenol compounds in biological samples [26], and information on the bioaccumulation of bisphenol compounds in fish, especially at the tissue level, is still limited. Because of the serious adverse effects of bisphenol compounds, it is necessary to develop a reliable and sensitive analytical method. Here, we developed an accurate and sensitive HPLC-MS/MS approach for the simultaneous measurements of BPA, and its alternatives BPS in six tissues from zebrafish, namely brain, gill, muscle, gonad, liver, and intestine.

Therefore, to gain a better understanding of the fate and potential toxicities of bisphenol compounds and MPs, the accumulation and tissue distribution of target chemicals, alone or in combination with MPs, were investigated in zebrafish. The results can not only help researchers understand the uptake and distribution of bisphenol compounds in the presence of MPs but also may provide deeper insight into their potential toxicity.

## 2. Materials and Method

### 2.1. Chemicals

The standard of BPA (CAS:80-05-7) (purity 99.8%) and BPS (CAS:80-09-1) (purity 99%) were purchased from Aladdin Biochemical Technology Co., Ltd. (Shanghai, China). Polyethylene microplastics with a particle size of 25 μm were purchased from Zhichuan Technology Co., LTD. (Jiangsu, Nantong, China). Methanol and acetonitrile were purchased from Thermo Fisher Scientific Inc. (Shanghai, China). All reagents were HPLC grade. The stock solutions of BPs (400 μg/L) were prepared by dissolving an appropriate amount of each standard in methanol. All solutions were stored at −20 °C until use.

Ammonium acetate, glacial acetic acid, and ammonium hydroxide (analytical grade) were provided by Beijing Chemical Co. (Beijing, China). β-glucuronidase from *E. coli* K12 was supplied by Roche Diagnostics GmbH (Mannheim, Germany). Ultrapure water was purified through a Milli-Q plus system (Millipore, Bedford, MA, USA). The SPE C_18_ cartridges were purchased from Meizheng Bio-Tech Co., LTD. (Rizhao, China). The 0.22 μm Filter Unit was from Bonna-Agela Technologies Co., Ltd. (Beijing, China).

### 2.2. Exposure Experiment and Sample Collection

Adult zebrafish (AB-wild type, aged 5 months) were purchased from the aquarium department of Hongdagaofeng and continuously cultivated in the laboratory for two weeks before the exposure tests (14 h light/10 h dark cycle, 25.0 ± 1.0 °C). During the acclimation period, the fish were fed two times daily, and rearing water was renewed every three days.

Adult zebrafish were randomly selected and exposed to different treatments, including a control (Ctr) group (zebrafish were exposed to dechlorinated tap water), BPA group (100 μg/L of BPA), BPS group (100 μg/L of BPS), MP group (100 μg/L of MPs), MA group (100 μg/L of BPA +100 μg/L of MPs), and MS group (100 μg/L of BPS +100 μg/L of MPs). The concentration of exposure (100 μg/L) was based on the environmentally relevant concentrations and could induce clear effects and identify possible mechanisms of toxicity [27,28].

Six replicates were set for each treatment, each of which contained 4 L liquid and 10 adult fish in a 5 L glass beaker. The solutions were changed every 3 days to ensure that the concentration of the tested substance was stable. The exposure period was 35 d. During the experiment, external conditions, including temperature, humidity, and light cycle, were consistent with the domestic environment.

After exposure of 35 d, fish were starved for 24 h, five zebrafish were collected randomly per replicate and anesthetized in MS 222 (Tricaine, Sigma-Aldrich, Shanghai, China). The muscle, brain, gill, gonad, liver, and intestinal tissues were removed quickly and set on ice. All samples were stored at −80 °C until extraction and analysis.

### 2.3. Sample Preparation

The zebrafish tissue samples were ground by a homogenizer, then 1 mL of 1 mol/L ammonium acetate buffer solution was added (pH 5.0, 7.71 g ammonium acetate dissolved in 93.4 mL of ultrapure water, 6 mL Glacial acetic acid, and 600 μL of β-glucuronidase). After the mixed solution was hydrolyzed in a 37 °C water bath for 12 h, 1 mL of methanol and 1 mL of water were added to the samples, thoroughly mixed with a refrigerated grinder for 10 min, extracted ultrasonically for 30 min, and centrifugated for 10 min at 10,000 rpm. The liquid supernatants were transferred to new 50 mL centrifuge tubes. The extraction described was repeated two times.

Samples were further purified with solid phase extraction (SPE) on a C_18_ cartridge which was preconditioned successively with 10 mL of methanol and 10 mL of water. After the sample was uploaded, the cartridge was eluted with 10 mL of methanol/water (5:95, *v*/*v*), and the elutant was discarded. The samples were then eluted with 10 mL of 5% ammonium hydroxide and were brought to dryness under a gentle flow of high-purity nitrogen and reconstituted with 1 mL of methanol/water (1:1, *v*/*v*). The final solution was filtered through a 0.22 μm syringe filter and then into individual 2 mL glass vials prior to instrumental analysis.

### 2.4. Analytical Conditions

Chromatographic analysis was performed on an ExionLC AE system (AB SCIEX, Framingham, MA, USA). A 22-min gradient on a HSS T3 column (2.1 × 100 mm, 1.8 µm) was employed for efficient separations. The composition of the mobile phase was acetonitrile (A) and 0.1% formic acid in water (B), with a flow of 0.3 mL/min and the following gradient: 0–1 min, 20% A; 1–13.5 min, 20–95% A; 13.5–18 min, 95% A; and 18–22 min, 20% A. The injection volume was set to 2 μL.

Mass spectrometry analysis was carried out on a SCIEX Triple Quad 4500 system with an electrospray ionization (ESI) probe in negative mode. The MS source conditions were as follows: curtain gas (CUR), 20 psi; collision gas (CAD), medium; nebulizing gas (GS1), 50 psi; heater gas (GS2), 60 psi; ion spray (IS) voltage, 4500 V; and source temperature, 500 °C.

### 2.5. Data Analysis

Statistical analysis and data illustrated by GraphPad Prism 10.2.0. Significant differences between groups were tested by one-way analysis of variance (ANOVA).

## 3. Results

### 3.1. UPLC-MS/MS Conditions

**Figure 1:**
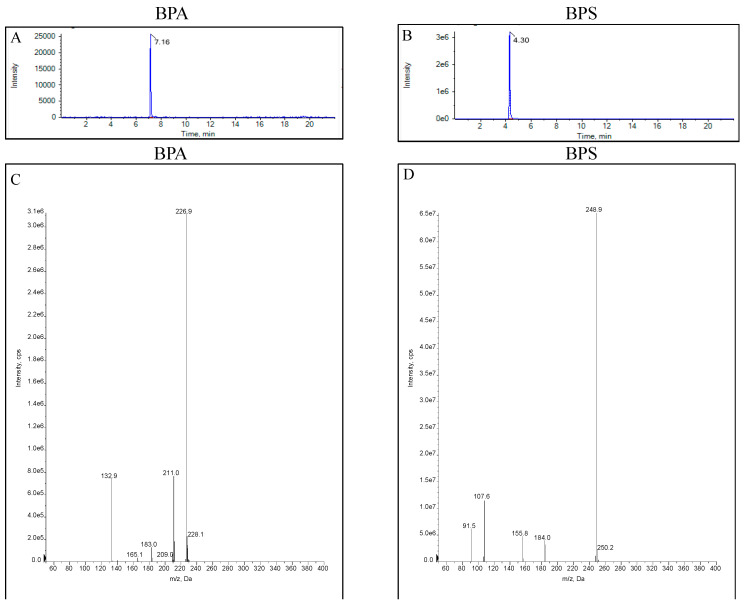
The UPLC-MS/MS chromatograms of BPA (**A**) and BPS (**B**) standards and their MS^2^ spectra pattern ((**C**): BPA; (**D**): BPS).

A BEH C_18_ column (100 mm × 2.1 mm, 1.7 µm) and HSS T3 columns (2.1 × 100 mm, 1.8 µm) were selected to separate the target compounds. However, it was found that BPS exhibited poor retention on the C_18_ column, resulting in rapid elution. In contrast, the T3 column provided better retention and separation for BPA and BPS, indicating a more suitable interaction with the stationary phase for the target compounds [29]. Then, the HSS T3 column was selected to analyze the targeted molecules. In preliminary experiments, methanol and acetonitrile at different concentrations were tested as the organic mobile phase. When acetonitrile was used as the organic phase, it had a better peak shape and peak broadening without obvious peak tailing. Furthermore, several studies have recommended that acidification of the mobile phase could improve sensitivity and ionization efficiencies, and therefore 0.1% formic acid was added to the water phase [30,31]. The sensitivity of both target compounds was higher than that of the pure water phase. The UPLC-MS/MS chromatograms of BPA and BPS standards and their MS^2^ spectra pattern were shown (Figure 1).

### 3.2. Sample Preparation

**Figure 2:**
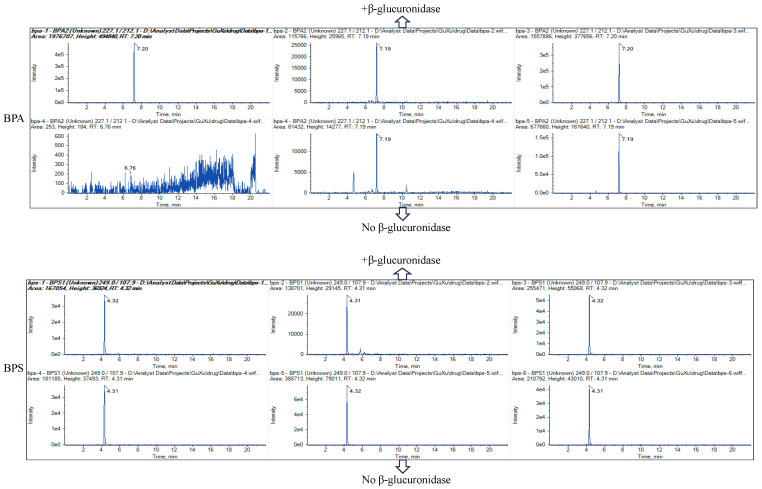
The chromatograms of extraction affect BPA and BPS with or without β-glucuronidase.

Regarding the widely different physicochemical properties of two target compounds, sample preparation is an important step to simultaneously extract all the analytes from a complex biological matrix. Acetonitrile, acidified acetonitrile (acetonitrile/formic acid = 99:1, *v*/*v*), 75% acetonitrile, 75% acidified acetonitrile (75% acetonitrile/formic acid = 99:1, *v*/*v*), ammonia acetonitrile (acetonitrile/ammonia solution = 99:1, *v*/*v*), EDTA–Mcllvaine buffer solution (acetonitrile/EDTA–Mcllvaine buffer solution = 9:1, 8:2, 7:3, 6:4, and 5:5) and ammonium acetate buffer solution (pH 5.0) were tested to simultaneously extract BPA and BPS from zebrafish tissues. The recoveries of all these extract solvents are shown in Table 1. Therefore, ammonium acetate buffer solution (pH 5.0) generated better recoveries than the other solvents and was finally selected as the extraction solvent for further study. Ammonium acetate can serve as a stabilizing background electrolyte and could effectively dissolve and extract bisphenol compounds, improving extraction efficiency and accuracy [32]. Next, the extraction effects with or without β-glucuronidase were also compared. It was found that the presence of β-glucuronidase significantly reduced background noise (Figure 2), possibly because the enzymatic reaction led to the degradation of complex matrix components and minimized matrix effects. Furthermore, previous studies have demonstrated that bisphenol compounds usually have conjugated forms in animal samples. Analytical methods for determining bisphenol compounds in biological samples usually use enzymatic hydrolysis to convert the conjugated into their free forms [33]. Therefore, β-glucuronidase was used in this study to ensure better extraction efficiency.

### 3.3. Method Validation

Accuracy, precision, linearity, limit of detection (LOD), and limit of quantification (LOQ) were validated for the developed method. Accuracy and precision were expressed as recoveries and relative standard deviations (RSD), the recovery assay was determined by six replicates at three different concentration levels (Table 2). Satisfactory recoveries for BPA from 78.8 to 109.5% and from 72.9 to 113.2% for BPS were obtained from different zebrafish tissues, with RSD < 12%, indicating efficient extraction.

The linearity was studied using matrix-matched standard solutions in triplicate at eight concentration levels, as summarized in Table 3. The correlation coefficients of the calibration curves were all higher than 0.99. The LOQ of the BPA and BPS ranged from 0.6 to 3.0 μg/L and from 0.8 to 3.0 μg/L, respectively, which indicated that the proposed analytical method is highly sensitive. In this study, all of the matrix effects were also presented in Table 3. Different tissue samples showed different matrix-induced effects or matrix enhancement (−11.2–12.0% for BPA and −13.8–13.9% for BPS). The reason for this effect may be the complex composition and organic matter in biological samples, and the influence of some unknown compound on the ionization efficiency of these analytes [33,34].

### 3.4. Tissue Accumulation of Bisphenol Compounds in Zebrafish

**Figure 3:**
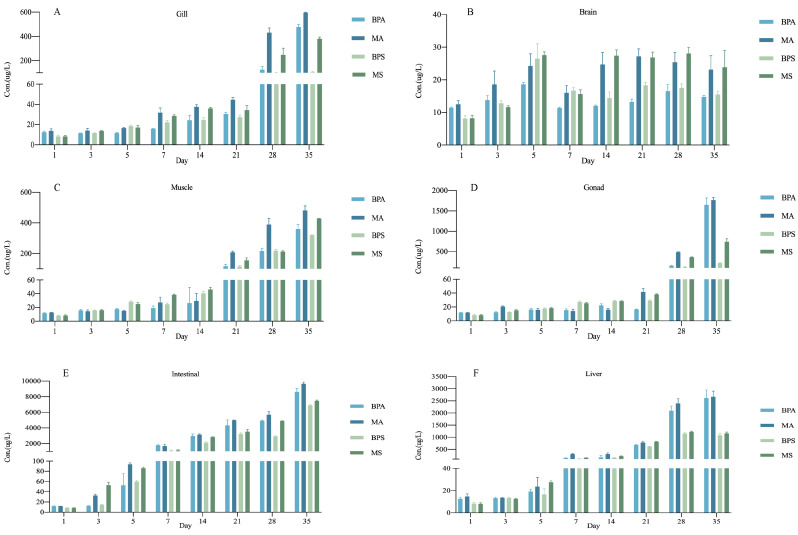
Tissue accumulation of bisphenol compounds with or without MPs in different zebrafish tissues. (**A**): gill; (**B**): brain; (**C**): muscle; (**D**): gonad; (**E**): intestine; (**F**): liver. The y-axis represents the accumulation concentration of bisphenol compounds in zebrafish tissues, while the x-axis indicates the different exposure durations. Error bars indicate the standard deviation. All values are represented as mean ± standard derivation (n = 3 biological replicated).

The concentration variation of bisphenol compounds in zebrafish tissues with and without MPs was presented (Figure 3). At all treatment levels, the bisphenol compounds concentration in various tissues gradually increased with time and the summed concentrations varied from tissue to tissue. Moreover, within the 35 d exposure period, the accumulation of BPA and BPS had not yet reached a steady state. These results indicate the considerable capacity of zebrafish to accumulate bisphenol compounds [35].

The concentrations of BPA and BPS in gill tissue were 12.38 μg/L and 8.28 μg/L on the first day, and these increased continuously during the accumulation period (Figure 3A). After 28 d, the concentration levels of BPA and BPS showed an obvious increase, and at 35 d, the concentration had reached 478.46 μg/L and 105.10 μg/L, respectively. In addition, the accumulation of BPA and BPS in gill was shown to be significantly boosted by the co-presence of MPs, which were approximately 596.11 μg/L and 381.08 μg/L after 35 d. We also detected bisphenol compounds in the brain (Figure 3B) and the concentration did not fluctuate at a large level (BPA: 14.76 μg/L; BPS: 15.47 μg/L), but the presence of MPs also lightly increased the accumulation of BPA and BPS (MA: 23.14 μg/L; MS: 23.84 μg/L).

In muscle (Figure 3C) and gonad (Figure 3D) tissues, the content of BPA was 360.10 μg/L and 1646.27 μg/L, respectively. The concentration of BPS in tissues was 319.94 μg/L and 215.19 μg/L at the end of exposure. Similarly, compared to a single exposure, the co-exposure of MA and MS showed more accumulation in muscle and gonad tissues (Muscle: MA was 481.55 μg/L and MS was 428.56 μg/L; Gonad: MA was 1767.04 μg/L and MS was 737.82 μg/L). The maximum accumulation of BPA/BPS was detected in the intestine (Figure 3E) (8624.61 μg/L and 6906.87 μg/L, respectively), followed by the liver (Figure 3F) (2620.23 μg/L and 1084.11 μg/L, respectively). Also, there were 9654.02 μg/L and 7457.46 μg/L with MPs in the intestine, 2674.18 μg/L and 1157.76 μg/L with MPs in the liver after 35 d exposure.

## 4. Discussion

### 4.1. Optimization of Sample Pretreatment

Three kinds of SPE cartridges, namely EMR-Lipid, HLB, and C_18_, were compared on their recoveries of bisphenol compounds in zebrafish tissues. EMR-Lipid is a unique absorbent that can be specifically adopted for the removal of lipids from biological samples [36,37]. EMR-Lipid was not suitable for extraction of BPA and BPS, with average recoveries lower than 60%, possibly because of the low-fat content of zebrafish tissues. Next to it was HLB, for which the target analytes were eluted during the wash procedure. This may be because of some hydrophilic functional groups in HLB columns [38], which were not suitable for the extraction of BPA and BPS in zebrafish tissues. The HLB cartridges have lower extraction efficiency for bisphenol compounds, as reported elsewhere [39,40]. C_18_ SPE cartridges provided good extraction efficiencies for both analytes (72.9–109.5%), which could be due to the functional group of octadecyl in the C_18_ cartridges. Compared with HLB, the functional group of octadecyl in C_18_ cartridges possesses a high carbon content and offers strong hydrophobic interactions [38]. Therefore, C_18_ cartridges are more suitable for the extraction of BPA and BPS in zebrafish tissues and were used in subsequent experiments.

### 4.2. Tissue Accumulation of Bisphenol Compounds in Zebrafish

At present, the tissue-specific distribution of pollutants in zebrafish is frequently observed and utilized to further investigate the mechanism of toxicity [41]. In our study, BPA and its BPS analog were detectable in zebrafish from the first day. Rapid uptake and quick accumulation of bisphenol compounds have also been reported [42,43]. In addition, the concentrations of bisphenol compounds steadily increased over the 35-day exposure period, without reaching a steady state, highlighting the potential for long-term bioaccumulation in aquatic organisms. The higher tissue concentration of BPA and BPS were in viscera, such as the intestine, liver, and gonad, more than in the gill, muscle, and brain. This distribution pattern likely results from xenobiotic transport processes and differences in lipid content among tissues, which play a crucial role in the bioaccumulation of hydrophobic compounds like bisphenols [43,44,45]. The liver, as the primary organ responsible for detoxification, is often a major site for the accumulation of xenobiotic compounds due to its role in metabolizing and excreting these substances [46]. The relatively high bisphenol concentrations found in this organ, as it acts as a central hub for chemical processing and storage, may explain the hepatotoxicity and disruption of lipid metabolism observed in response to bisphenol exposure [47,48,49].

As the initial absorption tissues, high accumulation levels of BPA and BPS were also found in the intestine. That means zebrafish could be also exposed to bisphenol compounds in the environment directly through ingestion. The intestinal tissue, being in direct contact with ingested bisphenols, acts as a primary site for accumulation before the compounds are distributed to other organs via the circulatory system [50]. Once bisphenols accumulated in the intestine, they could be transferred to hemolymph and then distributed to other tissues along with the circulatory system [35], as observed in the present study.

In contrast, tissues with lower lipid content, such as gill, muscle, and brain, exhibit comparatively lower accumulation of BPA and BPS. From direct contact with chemicals, fish could accumulate them from ambient water through respiration, suggesting the gills are priority organs for exposure [51]. Bisphenol compounds are difficult to metabolize coming through gills, resulting in deposition [46]. Fish gills have shown a significant accumulation of bisphenol compounds in the present study.

We speculated that the accumulation of bisphenol compounds in the brain further increased after co-exposure with MPs because its lipophilic chemical structure leads to bisphenols crossing over the blood–brain barrier [52] and reaching the fish brain along with blood circulation. The existence of BPA and BPS in the zebrafish brain might be the reason why bisphenol compounds could injure the nervous system [53,54]. The residue of BPA and BPS in the gonad may disrupt ovarian redox balance and oocyte health [55]. Early research made it clear that BPA and its analogs could induce reproductive toxicity in zebrafish [56,57]. The variation in bisphenol accumulation across tissues also underscores the importance of understanding tissue-specific toxicokinetics when evaluating the potential health risks posed by these compounds.

The results of this study revealed that while both BPA and BPS accumulated in various tissues of zebrafish, the accumulation levels of BPS were consistently lower than those of BPA across all tissues. This difference in bioaccumulation may be a key factor underlying their differential toxic effects. Structurally, BPS is one of the most common analogs of BPA, with a similar chemical structure that allows it to function in a similar manner in biological systems [58]. Despite this structural resemblance, several studies have reported that BPS generally exerts similar or lower toxicities compared to BPA [17,54,56,59]. The lower accumulation of BPS observed in this study could provide insight into these differing toxicological profiles. For instance, Boucher et al. (2016) and Moreman et al. (2017) both suggested that the reduced bioavailability of BPS may result in weaker endocrine-disrupting effects compared to BPA, which aligns with our findings of lower BPS tissue concentrations [60,61]. The fact that BPS does not accumulate to the same extent as BPA in zebrafish tissues, especially in key organs such as the liver and intestine, where maximum concentrations were observed, suggests that it may be less prone to long-term retention and bioaccumulation, which in turn could reduce its chronic toxicity. The noticeably lower accumulation levels of BPS compared to BPA might provide a theoretical basis for understanding their differential toxic effects.

Finally in our study, the co-exposure with MPs further facilitates BPA and BPS accumulation, especially in the gill, muscle, gonad, intestine, and liver tissues. These results suggest that MPs could enhance the bioavailability and uptake of bisphenol compounds, thus posing a greater risk to aquatic life. For tissue accumulation, previous studies have reported that the presence of MPs aggravates the bioaccumulation of environmental pollutants [13]. A series of recent studies revealed that the presence of MPs may disrupt the detoxification process in organisms [17,62], potentially contributing to the aggravated accumulation of BPA and BPS in zebrafish. Another plausible explanation is that MPs boost the accumulation of bisphenol compounds in different tissues of zebrafish through the Trojan horse effect [13,16,63]. The increased bioavailability and bioaccumulation of bisphenol compounds, facilitated by MPs, might be the reason why MPs can further aggravate their adverse impacts on neurotoxicity [43], immunotoxicity, neurotoxicity [64], and reproductive toxicity [16] of organisms. Given the widespread environmental presence of both MPs and bisphenol compounds, these findings have critical implications for the understanding of pollutant interactions and their combined effects on aquatic ecosystems.

Despite the significant findings, this study has limitations that must be acknowledged. First, while the accumulation patterns of bisphenol compounds were observed over 35 days, the lack of a steady state raises questions about the potential longer-term accumulation and effects beyond this period. Future studies should extend the exposure period to determine whether a steady-state concentration of bisphenol compounds can be reached and assess the chronic effects of prolonged exposure.

## 5. Conclusions

In this work, the development of a sensitive and accurate UPLC-MS/MS method for the simultaneous determination of BPA and BPS was described. This study is the first attempt to simultaneously determine two kinds of bisphenol compounds in different biological tissues. Good validation parameters were obtained in this work, laying a solid foundation for further analysis of the bioaccumulation and tissue distribution of bisphenol compounds in zebrafish. The developed method was successfully applied for the analysis of real zebrafish tissue samples. At the same time, the tissue distribution and accumulation of BPA and BPS alone or in combination with MPs in zebrafish were investigated. We found that the accumulation concentration of BPS is lower than that of BPA, which might provide a theoretical basis for the understanding that BPS has lower toxicity compared to BPA. More importantly, the results revealed that the copresence of MPs could aggravate the accumulation of BPA and BPS in different zebrafish tissues. Thus, further investigation on the potential risks of co-exposure of MPs and environmental pollutants to organisms and its underlying mechanisms of toxicity should be conducted.

## Figures and Tables

**Table 1:** The recoveries of BPA and BPS in all these extract solvents. (n = 3).

	BPA	BPS
Extract Solvents	Recoveries (%)	Recoveries (%)
Acetonitrile	72.6 ± 4.2	62.2 ± 15.6
Acidified acetonitrile	84.0 ± 1.9	66.0 ± 2.7
75% acetonitrile	68.3 ± 6.2	67.9 ± 5.8
75% acidified acetonitrile	69.7 ± 1.4	62.3 ± 2.9
Ammonia acetonitrile	75.7 ± 8.5	54.4 ± 16.6
Acetonitrile: EDTA–Mcllvaine = 9:1	79.5 ± 6.8	62.7 ± 11.1
Acetonitrile: EDTA–Mcllvaine = 8:2	75.8 ± 2.4	70.3 ± 3.2
Acetonitrile: EDTA–Mcllvaine = 7:3	45.4 ± 3.1	39.2 ± 9.2
Acetonitrile: EDTA–Mcllvaine = 6:4	34.5 ± 2.3	37.6 ± 3.0
Acetonitrile: EDTA–Mcllvaine = 5:5	50.9 ± 10.1	54.2 ± 1.1
Ammonium acetate buffer solution	98.5 ± 4.5	92.7 ± 0.8

**Table 2:** Spiked average recoveries and relative standard deviations (RSDs) of BPA and BPS in different zebrafish tissues. (n = 6).

Tissues	Spiked (μg/L)	BPA				BPS			
		Intra-Day		Inter-Day		Intra-Day		Inter-Day	
		Recovery (%)	RSD	Recovery (%)	RSD	Recovery (%)	RSD	Recovery (%)	RSD
Brain	5	78.8	8.2	89.2	8.7	81.6	3.6	72.9	11.1
	10	83.7	4.3	90.3	5.8	84.3	1.8	86.8	4.85
	20	83.9	5.5	91.2	3.9	79.6	4.5	89.7	8.0
Gill	20	92.6	5.2	92.4	7.1	96.5	2.9	95.6	1.9
	50	89.2	3.8	91.2	6.7	96.2	3.2	96.7	3.4
	100	93.8	3.6	84.6	3.3	94.8	2.9	89.0	6.2
Muscle	20	96.8	4.1	95.8	2.9	99.1	3.6	105.2	8.4
	50	94.1	5.3	95.4	4.2	86.4	1.1	83.9	1.8
	100	90.5	1.8	86.8	5.7	113.2	7.8	83.2	7.5
Gonad	20	91.4	5.9	93.2	9.1	98.2	7.9	84.1	6.1
	50	86.9	7.2	84.1	7.5	95.6	7.4	92.5	6.2
	100	87.6	1.4	84.6	5.2	92.9	6.3	93.8	4.8
Liver	20	96.5	9.2	94.9	6.8	94.8	4.2	84.6	9.7
	50	99.1	6.7	96.5	5.5	97.3	6.5	101.4	5.6
	100	101.3	11.1	93.6	3.8	97.1	9.8	95.6	11.9
Intestine	20	98.6	3.0	101.9	8.0	99.3	8.8	104.6	5.8
	50	89.7	6.6	97.1	9.8	95.8	10.1	95.6	9.0
	100	109.5	5.6	86.9	6.3	90.0	5.3	92.1	3.2

**Table 3:** Analytical performance of the UPLC-MS/MS method for different tissues.

BPA					
Tissues	Linearity Range (μg/L)	Correlation Coefficient (r)	Limit of Detection (μg/L)	Limit of Quantitation (μg/L)	Matrix Effect (%)
Brain	5.0–50.0	0.9984	0.3	1.0	5.6 ± 2.1
Gill	5.0–500.0	0.9994	0.3	1.0	8.8 ± 2.4
Muscle	5.0–500.0	0.9999	0.2	0.6	5.2 ± 1.6
Gonad	5.0–2000.0	0.9997	0.2	0.6	−9.4 ± 3.8
Liver	5.0–2500.0	0.9989	0.3	1.0	12.0 ± 2.7
Intestine	5.0–10,000.0	0.9992	1.0	3.0	−11.2 ± 3.5
**BPS**					
**Tissues**	**Linearity Range (μg/L)**	**Correlation Coefficient (r)**	**Limit of Detection (μg/L)**	**Limit of Quantitation (μg/L)**	**Matrix Effect (%)**
Brain	5.0–50.0	0.9990	0.3	1.0	13.9 ± 4.2
Gill	5.0–500.0	0.9992	0.3	1.0	5.7 ± 1.7
Muscle	5.0–500.0	0.9999	0.3	1.0	8.3 ± 1.1
Gonad	5.0–1000.0	0.9989	0.26	0.8	−4.8 ± 3.0
Liver	5.0–2000.0	0.9998	0.3	1.0	−15.6 ± 6.9
Intestine	5.0–10,000.0	0.9993	1.0	3.0	−13.8 ± 5.2

## Data Availability

Data available on request.
